# Designing and applying technology for prevention—Lessons learned in AEQUIPA and its implications for future research and practice

**DOI:** 10.3389/fpubh.2022.832922

**Published:** 2022-10-19

**Authors:** Jochen Meyer, Tiara Ratz, Alexander Pauls, Sandra Hellmers, Susanne Boll, Sebastian Fudickar, Andreas Hein, Jürgen M. Bauer, Frauke Koppelin, Sonia Lippke, Manuela Peters, Claudia R. Pischke, Claudia Voelcker-Rehage, Hajo Zeeb, Sarah Forberger

**Affiliations:** ^1^OFFIS – Institute for Information Technology, Oldenburg, Germany; ^2^Jacobs University Bremen, Bremen, Germany; ^3^Section Technology and Health for Humans, Jade University of Applied Sciences Wilhelmshaven/Oldenburg/Elsfleth, Oldenburg, Germany; ^4^Department of Health Services Research, Assistance Systems and Medical Device Technology, Carl von Ossietzky University, Oldenburg, Germany; ^5^Center for Geriatric Medicine and Network Aging Research, Heidelberg University, Heidelberg, Germany; ^6^Leibniz Institute for Prevention Research and Epidemiology – BIPS, Bremen, Germany; ^7^Institute of Medical Sociology, Centre for Health and Society, Medical Faculty, Heinrich Heine University Duesseldorf, Duesseldorf, Germany; ^8^Institute of Sport and Exercise Sciences, University of Muenster, Muenster, Germany; ^9^Institute of Human Movement Science and Health, Chemnitz University of Technology, Chemnitz, Germany

**Keywords:** prevention, technology, physical activity, older adults, intervention, interdisciplinarity

## Abstract

Almost all Western societies are facing the challenge that their population structure is changing very dynamically. Already in 2019, ten countries had a population share of at least 20 percent in the age group of 64 years and older. Today's society aims to improve population health and help older people live active and independent lives by developing, establishing, and promoting safe and effective interventions. Modern technological approaches offer tremendous opportunities but pose challenges when preventing functional decline. As part of the AEQUIPA Prevention Research Network, the use of technology to promote physical activity in older people over 65 years of age was investigated in different settings and from various interdisciplinary perspectives, including technology development and evaluation for older adults. We present our findings in three main areas: (a) design processes for developing technology interventions, (b) older adults as a user group, and (c) implications for the use of technology in interventions. We find that cross-cutting issues such as time and project management, supervision of participants, ethics, and interdisciplinary collaboration are of vital importance to the success of the work. The lessons learned are discussed based on the experiences gained in the overall AEQUIPA network while building, particularly on the experiences from the AEQUIPA sub-projects TECHNOLOGY and PROMOTE. Our experiences can help researchers of all disciplines, industries, and practices design, study and implement novel technology-based interventions for older adults to avoid pitfalls and create compelling and meaningful solutions.

## Introduction

In light of the current demographic shift and the consecutive increase of age-associated diseases, prevention and health promotion are key strategies in health research and practice. Community-based and individual prevention aims to prevent health impairments *via* targeted interventions, delay their onset, and mitigate the effects of pre-existing health conditions (1). Facing the surge of non-communicable diseases (NCD) (2), health promotion strategies that aim to empower people to increase control over their health behavior (behavioral prevention), e.g., by strengthening health literacy to reduce NCD risk factors, are seen as one pivotal approach toward healthy aging. In recent years, the rapid development and increased use of digital health technologies, such as wearables, dietary tools, and health and physical activity smartphone applications, have become highly relevant for health promotion (3–5). In addition to wearable technologies, ambient sensors have been used to analyze and promote activity-related indicators (6).

Despite the benefits of these tools, such solutions are often abandoned due to quality issues (7). In particular, sampling clinically relevant parameters with a high sensitivity and test-retest reliability by non-research-grade end-consumer devices, such as activity trackers (8), apps, and smart-home installations (9), is still challenging. Continuous reliability is essential for ensuring adequate quality and forms the basis for personalizing interventions and preventive measures, which, in turn, influence acceptance and usability (7). Therefore, interdisciplinary and participatory development and evaluation of technologies are necessary to design applications providing accurate data and promising high user compliance.

In the prevention research network AEQUIPA, eleven partners - universities, research institutes, and regional partners from disciplines such as public health, geriatrics, sports sciences, psychology, computer science, urban planning, and engineering-joined forces to tackle challenges in the promotion of physical activity among older adults by investigating the role of technology in this field. After more than 6 years of collaborative research, we present lessons learned and challenges faced when following a multidisciplinary and participatory approach that rigorously combines technological considerations with public health intervention design. These findings are based on the review of theories and evidence as well as co-creative work with representatives from the target population to enable co-design, followed by a series of internal meetings where the involved scientists came together to discuss and document their views on the potential of technology for prevention. In this discussion, three major areas evolved that we perceived as the overarching themes: (a) Design processes for technology-intervention development, (b) older adults as a user group, and (c) implications for technology use in interventions. Further, the paper highlights the great potential of an open and collaborative interdisciplinary approach to public health interventions and touches on several other relevant issues. The lessons learned will be discussed based on the experiences of the AEQUIPA network project. This paper aims to give an overview and summary of our findings to help researchers of all disciplines, industries, and practices design, study and implement novel technology-based interventions for older adults to avoid pitfalls and create compelling and meaningful solutions. An in-depth discussion of the respective topics is beyond the scope of this paper but can be found in the papers that we cite. Our experiences can help researchers, industries, and practitioners of all disciplines who are involved in designing, studying, and implementing novel technology-based interventions for older adults to avoid pitfalls and create compelling and meaningful solutions.

### AEQUIPA: Physical activity and health equity: Primary prevention for healthy aging

AEQUIPA (10) is an interdisciplinary regional prevention research network comprising seven universities, two research institutes, the health economy organization of the Bremen-Oldenburg metropolitan region, and the municipality Ritterhude. Since 2015 the AEQUIPA prevention research network has been funded by the Federal Ministry of Education and Research (AEQUIPA I 2015–2018, AEQUIPA II 2018–2022). The network's primary mission is to tackle the key challenges in promoting physical activity among older adults (65+). To that extent, AEQUIPA took a broad approach, investigating (a) the evidence-base for physical activity, (b) the environmental, contextual, and individual conditions enabling physical activity interventions, (c) the strategic linkage of urban planning and public health strategies, (d) new approaches for understanding health equity, and (e) the role of new technologies. Drawing on the ecological model of active living (11), the AEQUIPA network developed an interdisciplinary methodological design, including quantitative and qualitative studies, while combining different disciplines.

To understand the role of new technologies for prevention, three different types of technology were designed, covering different settings in prevention, addressing a range of research questions, and deploying a multitude of methodologies in multiple sub-studies (see Table 1 for more details about the sub-studies).

**Table 1:** Overview AEQUIPA technology and sub-studies covered within the article.

**Technology**	**Study name**	**Aim**	**Target group**	**Sample size**	**Research design**
Promote web-based intervention technology	Promote I	Compare the effects of two web-based interventions on physical activity compared to a delayed intervention control group	Age 65–75	589	Controlled intervention trial, testing two 10-week interventions with baseline and 12-week assessment
	Promote II	Compare the effects of two interventions using different modalities (web-based vs. print-based) on physical activity	Initially inactive older adults Age 60+	242	Randomized cross-over intervention trial; 10-week intervention and 24-week follow-up phase after cross-over option
ActiThings motivation toolkit	ActiThings	User Experience and effectiveness of ambient reminder technology	Age 65+, at least moderate technical affinity, inactive, intender	19	Comparative 3-month household study
	Interview study*	Needs and requirements for preventive technologies	Age 65+	33	Guided (semi-structured) interviews
	Difficult Target Group Analysis*	Needs and acceptance of preventive technologies, ActiThings toolkit, and assessment technology	Age 65+	27	Guided and structured focus groups
Versa Assessment technology	VERSA	Predictors for functional decline, development of technology-supported assessments	Age 70+	251	Observational study, three assessments over 2 years
	TUMAL	Development of measurement system for unsupervised standardized assessments	Age 70+, participants of the previous VERSA-study	91	Two assessments over 6 months plus monthly unsupervised assessments with the measurement system
	TUSMAL	Development of measurement system for unsupervised standardized assessments	Age 65+	About 30	Monthly unsupervised assessments with a measurement system freely accessible in a sports club
	TUMAL + TUSMAL	Needs & requirements, acceptance, usability, user experience	Age 65+ or 70+	57	Guided Telephone interviews + focus groups

*The studies marked were preliminary studies of the ActiThings motivation toolkit and Versa Assessment technology.

**Figure 1:**
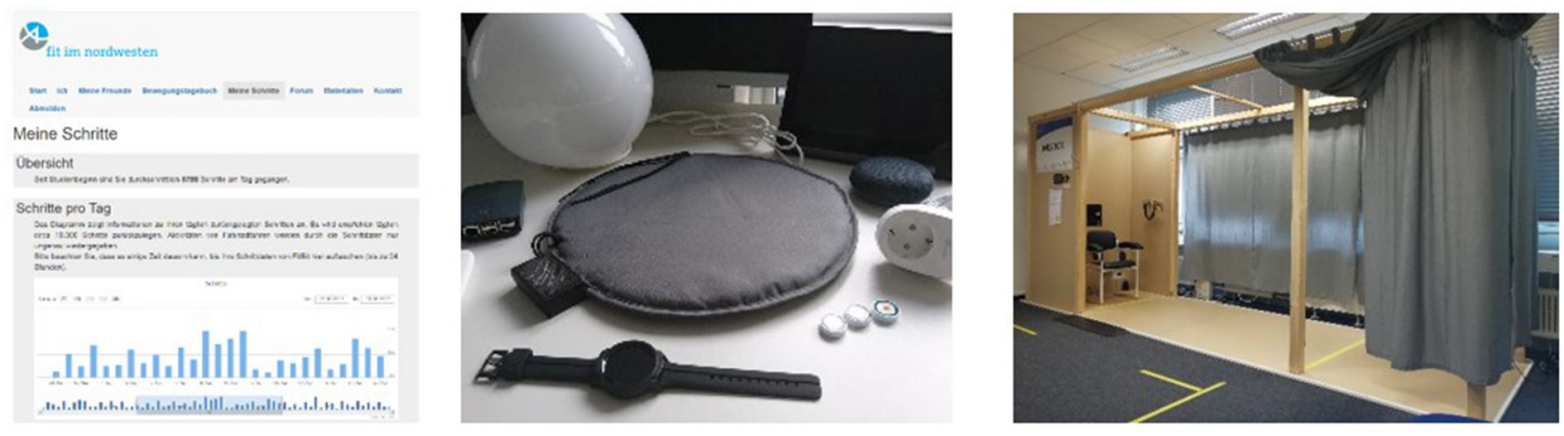
The novel technologies evaluated in **(left)** the PROMOTE-intervention-study– the website presenting the participants' daily activities, (center) the ActiThings motivation – interactive devices such as a tablet pc, pressure sensitive cushion, smart watch, plugs, lights and speakers that monitor the participants' behavior and remind of physical activity, and **(right)** the VERSA assessment studies – a measurement box equipped with multiple devices for unsupervised conduction of standard geriatric assessments.

**PROMOTE** (subsequently also: PROMOTE-intervention-study) aimed to understand the effects of web-based and wearable technology in a 10-week program for the initiation and maintenance of regular physical activity of older adults (12). The first PROMOTE study used an expert-driven approach to develop a web-based physical activity promotion intervention. A study with 589 older adults aged 65–75 years compared the effects of a web-based intervention with a web-based intervention plus activity tracker use on physical activity and with a delayed intervention control group. In the second PROMOTE study, the web-based interventions were adapted based on experiences gained in the first iteration. An additional print-based intervention with similar content to the web-based interventions was newly developed (Figure 1, left). In the subsequent study, the effectiveness of both interventions for promoting physical activity was compared in 242 initially inactive older adults aged 60+.

**ActiThings** (subsequently also: ActiThings-motivation) tested technological approaches for an integrated everyday physical activity program (13). Prototypes for technologies to remind of physical activity were designed and implemented in a user-centered design approach with end-users and experts (14) and pilot-tested in a workplace environment (15). The reminder technologies (Figure 1, middle) were evaluated for usability and user experience in a comparative 3-month household study with 19 inactive intenders age 65+ of at least moderate technical affinity. An interview study (16) and a hard-to-reach-populations analysis were conducted beforehand to allow for the incorporation of the target group-specific needs into the technologies.

**VERSA** (subsequently also: VERSA-assessment) developed a novel technology for the regular unsupervised conduction of geriatric assessments and tested its effectiveness in detecting early functional decline. A technology-supported geriatric assessment with high-end wearable and ambient sensors was developed in the first state. We focused on whether sensors can deliver the same results as conventional assessments and tested the effectiveness in detecting a functional decline in a 2-year observational study with 251 seniors aged 70+ (6). In the follow-up study TUMAL/TUSMAL, we developed a novel technology for the regular unsupervised conduction of geriatric assessments. A “measurement box” (Figure 1, right) was designed to deploy a broad range of high-end technologies such as a laser scanner and lab-grade accelerometers to facilitate the self-administered conduction of basic geriatric assessments such as the Timed Up and Go or the Five Time Chair Rise (16).

The article presents key lessons learned and challenges faced when following a multidisciplinary and participatory approach and rigorously combining technical considerations with public health intervention design. Pointing out prerequisites and success stories, pain points, and failures, these lessons can be helpful to researchers of all disciplines involved in developing, implementing, and evaluating novel, technology-based interventions for and with older adults, but also for industry and practitioners aiming to apply technology. They can also help politicians and policy-makers understand the challenges of working and collaborating in this field.

We group our experiences into three themes: the processes and methods involved in the interdisciplinary, user-centered, and iterative design, challenges and approaches when working with older adults as a target group, and implications for the appropriate technology design for older adults.

## Theme 1: Design processes

Technology-based interventions require appropriate processes and methods that build on and adapt established development procedures. In this section, we discuss the need for and implications of an interdisciplinary approach, elaborate on the role the user can take up in the design process, and refer to the influence of corresponding boundary conditions.

### Interdisciplinary design

Developing technology-based interventions requires expertise from multiple disciplines, including medicine, public health, social sciences, sports science, computer science, and psychology (17). These disciplines differ in their perspective, scientific traditions, vocabularies, concepts, and expectations for project outcomes. This makes the collaboration challenging, requires the willingness for mutual learning, and takes time and resources. Yet, when these challenges are dealt with appropriately, interdisciplinary research provides excellent opportunities to broaden the views of those involved and disseminate genuinely innovative results.

#### Technology- vs. application-driven design approach

Interdisciplinary collaboration between technology- and behavioral intervention developers can cause researchers to choose between two opposing perspectives: a technology-driven approach vs. an application-driven approach. In a technology-driven approach, the behavioral intervention includes a piece of technology developed first, and the intervention is designed around this technological component. This may lead to a new intervention, but the results may not meet precisely the needs of the users. It may not be accepted or may not make sense at all when being put into practice. In contrast, in an application-driven approach, the intervention is designed and planned first, and the supporting technology is integrated later on, with the aim of specifically matching the intervention's purpose. This bears the risk that the potential of new technologies may not be fully exploited, and the intervention may not be as good as it could be. If the specific requirements of the selected technology cannot be adequately met, the results may be unsatisfactory and may not work as intended.

#### Potentials and challenges of a co-design approach

A possible solution for combining both approaches could be the co-design approach (18), where the needs and possibilities of both the intervention and the technology are aligned in a creative, iterative process. In this case, an interdisciplinary way of working is used, where all stakeholders contribute with their expertise, learn from each other and create something new. This co-design takes time and effort and may require multiple iterations of development. In addition to finding and establishing a common vocabulary and underlying concept for the applied terms, this approach also poses a challenge to traditional study approaches that prioritize an early established study design which is not dynamically adapted in most cases. In contrast, the co-design approach requires careful planning and preparation and an interdisciplinary dialogue involving all stakeholders when the study design needs to be adapted.

#### Experiences from co-design approaches

In AEQUIPA, different co-design approaches were chosen. Within the network's first 2 years, there was an extensive exchange of approaches, concepts, understanding of terms, working philosophies, and legal frameworks that comprehensively integrated the knowledge of technological and intervention-based approaches across the network. This resulted in a diversity of implemented design processes: In the PROMOTE-intervention-study, an existing intervention was adapted to integrate off-the-shelf technology. In ActiThings-motivation, novel technologies, as well as an intervention scenario, were developed from scratch. And in the VERSA assessment, well-established geriatric assessments were the basis for developing new technological approaches adapted to match technical opportunities.

### User-centered design

#### Potentials and challenges of user involvement

The users are an integral and essential part of the interdisciplinary process in AEQUIPA. User involvement in designing interactive systems has been considered good practice for many years (19). Evolving from the social sciences, it is also now frequently applied in the design of interactive technical systems, and numerous variations of user-centered and participatory design processes have been suggested. Participatory design is “an approach to design attempting to actively involve all stakeholders … in the design process to help ensure the result meets their needs and is usable” (20). According to Kyng (21), the central aspect of participatory design is to engage potential user representatives willing to collaborate with researchers to produce new technological systems that are easy to understand and use. Hence, it can be helpful to follow this design approach to gain insights into views, and first-hand experiences of the target group on specific interventions or offers on the topic, as well as to learn about the experiences of experts working with the target groups.

However, in practice, user involvement may be challenging. It requires financial and human resources and takes time. For example, given the heterogeneity of the target group concerning, e.g., age, health and digital literacy, and physical and mental health state, it can be challenging to address and reach all relevant viewpoints. In more technical developments, user involvement does not cover all relevant aspects, resulting in the necessity of the use of other approaches, such as human-centered design (22), design thinking (23), or theoretical framework for user involvement (24).

#### Implementation of user involvement in AEQUIPA

In preparation for the development of ActiThings–motivation and VERSA-assessment technologies, three focus groups were conducted with different groups (e.g., people with migration backgrounds, non-technology-affine people, and significantly older people) as part of a target group analysis. The surveys focused on questions about the favored devices for ActiThings, suggestions for improvements to the menu, and VERSA instructional videos. These findings were subsequently integrated into the prototype development.

Therefore, VERSA followed a stepwise participatory design process with two development and evaluation cycles. The first usability study consisted of three parts and evaluated the prototype with a group of 10 people: (1) general introduction, (2) task-based evaluation, and (3) guided interviews. An evaluation prototype was then developed based on these findings. In a final evaluation study, relevant experiences and suggestions for further improvements were derived from interim discussions based on guideline-based telephone interviews and focus groups with 37 participants (25).

To inform the design of the ActiThings-motivation toolkit, the initial basic design was extensively discussed with geriatricians and experts for medical devices. After that, a human-centered design approach with three iterations was conducted (13, 14). In the first stage, three conceptual designs were developed and discussed in two focus groups with five older adults. In the second iteration, functional prototypes were implemented that were tested individually by five older adults in an apartment-like lab in a roughly 1-h session. In the third and final iteration, the prototypes were refined based on the findings of the previous evaluations. Finally, a field usability test was conducted with six older adults from five households in their homes.

The development of the PROMOTE-intervention-study's technology took a combined expert and end-user-driven approach to create a medium-scale intervention study (26). The rationale for involving potential intervention participants and other stakeholders (e.g., experts in the field of physical activity promotion) in developing the second iteration of the intervention and study was to reach predominantly inactive older adults. Hence, intervention messages and content of the technology-based intervention material had to be modified to fit the needs and preferences of the primarily inactive target group and prevent attrition. Workshops and focus groups were held with members of the target group of older adults and experts in the field of physical activity programs targeting older adults, and potential multipliers of intervention messages and materials (stakeholders in communities: members of senior citizen organizations, advisory boards) (27). Results ranged from recommendations regarding font size and color, amount of text to read to the appropriateness of the content and aspects to consider regarding the target group. All suggestions were used for developing the material from the first to the second iteration while also implementing a preference design: participants were randomized to use either print or digital material. They could, after 3 months, choose whether they wanted to continue with the given modality or to switch to the other modality (27). While it was expected that study participants would have balanced preferences for print and digital material, and individual characteristics would determine the preference (27), the observation was that relatively few study participants decided to change modality (28). Change preference was more frequent in the print group (15 of 91 individuals preferred to continue with the digital mode) than in the digital group (only one out of 104 individuals referred to change to print). However, dropout was more likely in the digital group [“35.2% in the web-based intervention group, 42.1% in the web-based intervention group including a pedometer, and 30.1% in the print-based intervention group” (28)].

### Iterative design

#### Challenges of developing technology prototypes for a study context

Developing systems for evaluation and study purposes often causes a dilemma for researchers. Users may already have experienced professional apps and expect similarly high reliability and high quality of the new system. However, given that system development is a highly professional process with great efforts, research prototypes can by no means be similarly polished. But even in a study context, ambiguous displays and interactions or potential malfunction of the systems may not be tolerated by study participants, which may heavily affect the outcomes of a study. The possible best solution is to follow an iterative design process. Such a process has been well established and offers excellent paths from initial design concepts to a usable and acceptable interactive system (29).

#### Iterative design processes on the example of ActiThings

We applied it successfully in the design of the ActiThings toolkit. We started with early and technically relatively simple “low fidelity” prototypes that already included relevant and essential functions and are suitable for experimental studies but were not intended for real-life use. We use these to explore different options and understand various design opportunities. We evaluated those under controlled conditions in a lab setting and also with younger people – not the later group – to understand properties and user experience. In the next steps, we continuously increased the realism of study parameters toward a more natural setting in the real world. We evaluated each step, more and more also involving the later target user group, and it had to show significant results in experiments to be scientifically valid. We found that increasing this so-called ecological validity (30) requires many steps in which the design of the system, the target user group, and the target scenario and context are stepwise approaching the final target setting. This is a time-consuming and therefore costly approach. We managed this with the limited resources of a research project by concentrating on the core functionalities of the target system, leaving out any unnecessary functions outside our research questions. With this approach, we managed to design and implement a complex interactive technical system that could successfully be deployed in a three-month in-the-wild household study.

## Theme 2: Older people as a user group

The increasing proportion of older persons and their rising life expectancy affects traditional roles (extension of working life and leisure time, assumption of cross-generational care work). It leads to an expansion of the life phase (31). In addition to this expansion and changing role expectations, immigration also leads to increasing heterogeneity in old age (32). The pluralization of life courses and culturally different biographical arrangements enhance the heterogeneity of this life phase (33). It is characterized not only by high inter-and intra-individual variability in all dimensions but older adults also vary in technology use and preferences. Current data from a German study on internet use reveal that about 93% of older adults aged 60–69 years and 75% of older adults aged 70 years and above use the internet at least occasionally (34); whereas 57% (60–69 years) and 34% (70+ years) do so regularly. The use of the internet and technical devices depends, besides age, on several factors such as sex, socioeconomic status, and cultural background (34–36). However, external factors, such as pandemics, might influence technology use, as daily routines and preferences might change, e.g., because non-digital alternatives become less feasible (34). This heterogeneity must be considered when developing and designing tools or technical devices for older adults, as the different preferences will influence the use, acceptance, and support in everyday life and may cause a digital divide. As mentioned above, in preparation for ActiThings-motivation and VERSA-assessment technology, three focus groups were conducted with different heterogeneous target groups (people with migration backgrounds, non-technology-affine people, and very old adults), the results of which were incorporated into the development phase of these technologies. In PROMOTE, heterogeneous technological affinity was addressed by offering different intervention modes of delivery, ranging from print to web-based materials and wearables, in addition to a web-based intervention.

### Recruitment strategies

Besides developing user-centered technology, recruiting study participants unfamiliar with technology may pose a challenge. Various older groups, e.g., non-technical-affine people or people with a migration background, often require a targeted approach and detailed information. Simple flyers or press articles are insufficient to inform these people comprehensively about a study or motivating them to participate. Other strategies are then often necessary. To recruit study participants, passive (e.g., press relations, public events, flyers) and active strategies (e.g., personal invitation appeals, use of multipliers) can be used (37–39). A review of 47 included studies recruiting participants for walking interventions (e.g., older adults) suggests that passive strategies were more frequently used than active strategies. Few studies examined which strategies were effective in improving recruitment (39). In projects in the field of prevention, for example, multipliers from the target group helped create channels for recruitment (40).

#### Recruitment strategies deployed in AEQUIPA

Recruitment for VERSA assessment took place *via* sports clubs, senior citizens' meetings, rehabilitation sports centers, multiplicators, press relations, and a health insurance company (16). In ActiThings-motivation, participants were recruited through existing databases, flyers, and public relations activities, for example. Access based on the press can be evaluated most successfully (13). For an interview study in the same subproject, passive (press relations, flyers) and active strategies (community work staff at a neighborhood meeting place) were used. Especially through press relations, many people came forward. To reach older people with a migration background in a targeted manner, the direct approach by the community work staff at a neighborhood meeting place is to be evaluated positively as an active strategy (41). The PROMOTE intervention study also used both passive and active strategies and aimed to recruit primarily inactive older adults living in a mixture of rural and urban communities in the Bremen-Oldenburg metropolitan region. In PROMOTE I, the five communities with the lowest community readiness assessment score for implementing physical activity promotion programs targeted at older adults were chosen as recruitment sites (42). In the study participation invitations were sent *via* postal mail to random samples drawn from the residents' registration office, both PROMOTE intervention studies used language that targeted older adults with at least a basic knowledge of German who lived independently. The combination and tailoring of recruitment methodologies were intended to motivate a demographically diverse sample of the target group (including individuals with low SES, advanced age, and a non-German background) to participate (43). However, the PROMOTE-intervention study faced the problem of selection bias toward more active and healthier older adults without a migration background, as well as higher attrition in technologically non-affine subgroups, highlighting the need for appropriate recruitment strategies and participant support, especially over the longer term.

### Participant support

Technology-based interventions may require study participants to engage in the intervention in an unsupervised setting, such as their homes. The lack of immediate contact between study facilitators and participants requires professional support strategies to ensure intervention uptake and adherence (44).

#### Support for uptake of technology

Study participants risk discontinuing or being non-compliant if they are not taught how to use the technology in the intervention or whom to contact for technical support. This is especially relevant for individuals who are not affine to technology or are unfamiliar with it, which is quite common in elderly people. Uptake and adherence can be increased by providing technology training *via* introductory sessions and well-designed information flyers, which we used extensively in all three settings. These efforts require researchers to carefully evaluate resources to plan the setting, staff, duration, equipment, and frequency of participant support components. Immediate participant support, using a telephone hotline or contact email, supports users at home when encountering problems with technology.

#### Support for adherence over time

Furthermore, intervention engagement may change over time, potentially leading to a declining adherence to the technology-based interventions and dropout (45). Strategies such as telephone hotlines, regular messages or follow-up phone calls, personal meetings, greeting cards, or a feedback function for participants might be helpful strategies to prevent adverse effects. For example, the dropout rate in the first iteration of the PROMOTE intervention study was ~30% after 3 months, with the highest proportion in the group with the highest number of technology-based components (12, 43). In the second iteration, where technological affinity and mode of delivery preferences were addressed explicitly during intervention implementation, the dropout rate was only about 20% after 3 months and ~30% after 9 months (28). In ActiThings, where the researchers regularly checked the system's functioning and a phone and an e-mail hotline were installed, 10% of participants dropped out (13). The drop-out rate in the first VERSA sub-study was 9% after 24 months (16), and for the second sub-study (6), 2% after 6 months.

## Theme 3: Implications for technology-based interventions in older adults

The technology design for an intervention must take the characteristics of older persons as a specific target group into account. In the subsequent sections, we outline the characteristics of the user. Knowing the user groups and their needs greatly influences the requirements for the technology to be developed or used. There is an extensive and comprehensive body of research about requirements for technology for older persons, for example, in human-computer interaction (46) or medical informatics (47). We subsequently focus on selected aspects that we find particularly relevant in the context of our work.

### Barriers to access to technology

As new health care and prevention components, technologies require adaptations at individual and systemic levels. While there are no particular barriers to accessing health information and technologies for many older people of high socioeconomic status, public access to the health care system and also financial resources necessary for the implementation of technologies (internet connection, devices) may often not be available to people of lower socioeconomic status (35, 36). To prevent the exclusion of these groups, this information and these technologies must be accessible and affordable, and the health care system should support them.

Furthermore, integrating the use of technologies and the associated behavioral changes into daily life is relevant for the acceptance and sustainability of the interventions (48). Key aspects are: (i) a non-stigmatizing design of components, (ii) a simplification of interaction and support in the learning phase, and (iii) stability and data security of technologies, as users often interpret software errors as user errors.

To include as many groups as possible, we developed, for example, in VERSA, an assessment system (see Figure 1, right) for the community and installed it at publicly accessible and easily reachable places like a sports club or the university. We also provided on-site support during the opening hours to assist the users if necessary, especially in the learning phase or in the event of system errors. In ActiThings, where household technology was developed, identifying and removing or lowering barriers access to technology was a crucial aspect of the co-design process, resulting in a system that aimed to be as unobtrusive and as easy to use as possible.

Although many applications are designed as stand-alone systems for older people, the interconnectivity and usability of the technologies by healthcare providers are also relevant. Here, too, the reliability of the technologies (including the European General Data Protection Regulation, certificates according to Medical Device Regulation, and liability issues), as well as the ease of use, the introduction to use, and the integration into the IT structure are of high importance.

### Awareness of age-related functional decline in technology design

For the use of and interaction with technical devices, sensory, motor, and cognitive functions play a central role. However, sensory functions like hearing, vision, and touch decline with age (49). For example, concerning motor requirements, manual dexterity or finger control, as well as the sense of touch at the fingertips, are essential. The sense of touch and tactile perception decline begins in early to middle adulthood, accelerating from ~45 years onwards. From 70 years on, almost all older people are affected by changes in the sense of touch (50). These sensory changes need to be addressed in technology development. The decrease in tactile perception can lead to problems with routine fine motor movements, such as the operation of touchscreens or small input devices. When using a mobile phone, it should be ensured that the keys are sufficiently large and stable. Regarding the deterioration of the vision, appropriate font size, lighting, and color contrasts should be considered.

About the decline of cognitive function in older individuals, decreased attention, executive functions, and memory, as well as reduced information processing speed and accuracy, are the most commonly reported complaints in older people (51, 52) and should be considered during technology development. In general, there is a pattern according to which functions linked to processing speed and requiring a lot of resources tend to show losses. Fortunately, semantic and procedural memory remains stable or even increase over the life course. In many cases, older adults manage to compensate for lower performance in the abovementioned areas with expertise and strategies (53).

Therefore, we considered the cognitive and sensorimotor impairments in the development of the technology in the VERSA assessment by adapting the screen display (easily readable and large font, high contrast, simple presentation) and the instructions (simple, short, and precise information) (25). The PROMOTE app was designed to be straightforward to use, having only the minimum required functionalities, including large user interface elements and a simple screen design (43).

### Demand for instant feedback

#### The sufficient amount of feedback

Technology-based interventions for prevention often aim to change behavior by drawing attention to the current behavior or outcome – for example, using self-monitoring or feedback. PROMOTE used a web-based physical activity diary to support self-monitoring and fitness trackers to provide feedback on behavior. We observed that some older adults reacted adversely to external control mechanisms, such as feedback on physical activity. In contrast, some stated that they would require self-monitoring to assist them in reaching their physical activity goals. These observations are corroborated by previous findings showing that promoting self-monitoring and providing feedback on behavior may lead to decreased physical activity or self-efficacy in older adults (54). Other subgroups of older adults may appreciate and benefit from external, technology-assisted support (55). Feedback on outcomes could increase adherence by visualizing health improvements, which can be particularly relevant in prevention studies (56). Thus, whether and how giving feedback must carefully be adapted to the individual needs.

#### Reasons for giving feedback

ActiThings foresaw no monitoring whatsoever due to regulatory concerns. Instead, we observed that participants developed their own self-monitoring and feedback mechanisms. We identified at least three reasons why giving feedback is essential (13): those involved in preventative interventions have high interest in health, may have a perceived higher health risk, and want to take control of their health. Feedback confirms that users interact correctly with the technology. And feedback increases perceived control over and trust in technology. Thus, intervention mechanisms should be designed considering how technology-assisted tools are perceived by the target group – and should possibly even be individually tailored. Feedback should be given just-in-time (e.g., about performances today or confirmations of actions taken). It also should be given in the mid- and long-term (e.g., for reviewing changes over time or facilitating reflection on performance and success). The VERSA assessment only provided feedback on the successful or incorrect performance *via* the assessment system. The medical evaluation of the test performance and the changes over time were explained for regulatory reasons in personal interviews with medical experts at the end of the study.

### The adequate level of tailoring

Tailoring involves personalizing an intervention to individual needs, resources, barriers, prior knowledge, motivation, or experiences. It is the opposite of a “one size fits all” approach (57), and there is scientific evidence that tailoring the interventions increases the length of participation [e.g. (57–59)]. Technology provides unique opportunities for tailoring and allows for a high degree of individualization through customization and “just in time” feedback. A normative database (e.g., evidence-based recommendations) and an ipsative database (previous information provided by the individual) can be used to guide and personalize intervention content (60).

In PROMOTE, technology components were not tailored to technological affinity, potentially limiting intervention effectiveness but leading to a simplicity that was easy to deal with both the researchers and the participants. In contrast, in ActiThings, the intervention was extensively tailored to participants' personal environments and daily lives. While it was generally well accepted, we also found that the possible variation added complexity to the intervention that some users had difficulties dealing with. Some users also wished for dynamic tailoring, evolving over time, as the intervention showed positive effects. On the other hand, in ActiThings, we observed that, after the initial set-up, users did not change the system's configuration (13). Similarly, in the second iteration of PROMOTE, most participants remained in their initially randomly assigned intervention group (28), indicating that persistence may be more important than flexibility. Therefore, we suggest that tailoring and adaptability should be carefully balanced with ease of use and predictability.

## Proposed actions and recommendations

Based on the findings presented above, we derive the recommendations for action presented below.

### Managing time as a scarce resource

Co-designing technology-based interventions take time, and time is a scarce resource in a research project. In the PROMOTE studies, we made some compromises in the design phase to leave sufficient time for conducting the study, such as not explicitly focusing on persons with low socioeconomic resources or with different language requirements. This had some adverse effects on study participation and results later on.

The combination of intervention research and technology development in the different co-design approaches allows for beneficial hands-on planning. However, sufficient time for feedback and iterative loops during the project must be planned. Therefore, studies with a robust technology-centered approach require more time for developing and testing prototypes, which need to be subsequently piloted in the interventions.

Older persons as a target group have specific needs, require time to explain the intervention, learn the technical functionalities, and reduce uncertainties. Studies targeting this group must be particularly carefully prepared, and well-designed information material is needed. Such resources must be planned appropriately. These aspects become even more critical when the introduction of technology to older persons is intended who tend to be often less technically affine. Initial technology training, ongoing supervision of potential critical issues, and in-person support for resolving problems are needed to successfully include older individuals in studies and minimize dropout rates. These requirements can lead to longer project durations and higher financial needs, particularly when testing technology in real-life settings.

Research projects should not underestimate this point and budget for these costs but also plan for dynamic controlling to quickly recognize deviations from the planning and to be able to take countermeasures so as not to endanger the course of the project.

### Managing and supervising the participants

Acceptance of technology in the target groups was very heterogeneous. While some participants appreciated technology and were willing to deal with it, others were highly critical. Some were unforgiving for even simple problems, or they generally rejected it for no specific reason, even if they reported a good technical affinity. This led to increased efforts in participant management, drop-outs, and a negative impact on the study results.

Drop-out in studies is also a general challenge. Despite careful supervision, participants decided to leave the studies or didn't attend scheduled meetings, again leading to a negative impact on the study results.

Future studies should consider this and include different considerations in the planning, for example, either by planning the participation management according to sufficient resources or by assessing the participant groups in terms of technology affinity and acceptance and disseminating the materials and the support structures according to the different needs.

During the project, we became aware of three other issues that touch on all of the areas mentioned in this article but were not explicitly addressed: interdisciplinarity, ethics, and project management. However, as they are essential for good projects, we have included them in the lessons learned.

### Interdisciplinarity as a challenge in collaborative research

The interdisciplinary structure of the research team made the work challenging, especially in the initial phase, as common approaches and understandings had to be established first. Technology development and intervention development follow their internal logic. These first had to be harmonized with one another. Even when the same terms were used, the underlying concepts sometimes varied greatly. These misunderstandings had to be recognized and resolved in discussions and cooperative interactions.

Interdisciplinary research projects need to schedule enough time and space for discussions. Joint workshops, glossary work, and publications can facilitate mutual understanding. Interactions must be structured and coordinated so that the respective media and the specific knowledge of different disciplines are symbiotically linked.

### Dealing with ethics

The study participants' safety is the highest priority when conducting a study. Thus, important issues regarding ethical aspects, data protection, and data sovereignty had to be considered in the technology studies of the AEQUIPA project. The responsible ethics committee approved the individual study concepts, and possible risks were analyzed and reduced as far as possible.

Generally, the fundamental ethical framework for using technical systems must be continuously considered during the research process (i.e., in the studies). In addition to general ethical values (61) and specific aspects in older people (62), the main focus of technological interventions for movement encouragement is the trade-off between short- and mid-term benefits on the one side and the risks of acute injury during exercise on the other side. In addition to the data on side effects to be routinely collected, attention must be paid to adequate information on potential risks to the subjects (comprehensive informed consent). Furthermore, the technological systems should have integrated some kind of health status recognition of the participating subject, or they should be able to obtain assessments from specialist personnel (dynamic risk assessment). In particular, health data collection and exchange play a significant role. Mechanisms for controlling the flow of information by the participant must be implemented for this purpose (data sovereignty). These aspects should also be included in future (commercial) products and services. Quality standards beyond pure functional safety must be developed, and close integration into care structures must be ensured.

### Project management and communication

Managing a multi-year, large-scale research project is a challenge in any case. We would like to emphasize a few aspects of our interdisciplinary approach, to our specific target group of older persons and our focus on technology. As pointed out earlier, the various disciplines involved have different working methods. We, therefore, put effort into getting to know each other and learning from each other starting during the project's first months up to its end. Project plenary meetings were essential to increasingly understand what others were doing, also providing a broader view beyond the personal project work. Clear communication strategies and a communication plan were developed, so that team members from different discipline's became aware of any misunderstandings and developed a common language, but also so that space was created for communication in the project. The precise distribution of tasks led to clear structures of responsibility and accountability, bringing a strong sense of responsibility and self-awareness with a high level of personal identification.

Projects above a certain size should critically consider using a project manager or a coordination team to manage the project coordinatively to ensure internal and external communication, coordination, networking, and controlling.

## Conclusions

Technology undoubtedly provides numerous opportunities for healthy aging. However, researching and developing interventions come with challenges and issues. We identified three main themes – the need to choose the appropriate design process, the need to consider the needs of older adults as the target group, and implications for the technology design. Furthermore, we found important tasks for project planning, participant support, interdisciplinarity, ethics, and project management. Not only are these challenging topics in themselves, but we found it necessary to tackle them jointly. Initially ignoring one or more of them will, according to our experiences, lead to problems at a later stage. Given the practicalities of research planning and the dynamics of research, we are aware that a perfect long-term project plan cannot exist. There are approaches to tackling these challenges. Still, finding the best solutions for a given problem remains a complex task that requires not just time and resources but also experiences and possibly a bit of luck. Nevertheless, we strongly encourage future research to be aware of all these issues from the very beginning and make plans about when and how to address them.

This article presents results from more than 6 years of research work. The results do not claim to be complete and comprehensive but merely document the points we found particularly challenging, important, or striking. While they are, to some extent, a subjective selection, they still represent the core items that a large and interdisciplinary group of experienced researchers agreed on as overarching themes. We know that other researchers have already documented many of our findings. Nevertheless, we believe that in its entirety and based on 6 years of interdisciplinary and collaborative research, our results provide a valuable addition to what has already been discussed before, providing a practical reference for dos and don'ts. By documenting our experiences, we, therefore, hope to inform subsequent research, industry, and practice and inspire policy-makers and politics and thus contribute to the success of future research and development of technology-based interventions for healthy aging.

## Ethics statement

The ActiThings and VERSA studies involving human participants were reviewed and approved by CvO University. Oldenburg Medical Ethics Committee No. 2018-046 CvO University. Oldenburg Medical Ethics Committee No. 2018-143. Hannover Medical School No. 6948. The PROMOTE I study was approved by the Ethics Committee of the Chemnitz University of Technology (TU Chemnitz), Faculty of Behavioral and Social Sciences (number: V-099-17-HS-CVR-PROMOTE-03072015). For the PROMOTE II study, ethical approval was obtained from the Medical Association in Bremen (RA/RE-635, on 3 July 2018). All patients/participants provided their written informed consent to participate in this study.

## Author contributions

All authors listed have made a substantial, direct, and intellectual contribution to the work and approved it for publication.

## Funding

This research is funded by the German Federal Ministry of Education and Research (AEQUIPA I and AEQUIPA II, Project No. 01EL1822A, 01EL1822C, 01EL1822D, 01EL1822E, 01EL1822F, 01EL1822I, and 01EL1822J).

## Conflict of interest

The authors declare that the research was conducted in the absence of any commercial or financial relationships that could be construed as a potential conflict of interest.

## Publisher's note

All claims expressed in this article are solely those of the authors and do not necessarily represent those of their affiliated organizations, or those of the publisher, the editors and the reviewers. Any product that may be evaluated in this article, or claim that may be made by its manufacturer, is not guaranteed or endorsed by the publisher.
